# Pharmacokinetics and biodistribution of Erufosine in nude mice - implications for combination with radiotherapy

**DOI:** 10.1186/1748-717X-4-46

**Published:** 2009-10-23

**Authors:** Guido Henke, Lars H Lindner, Michael Vogeser, Hans-Jörg Eibl, Jürgen Wörner, Arndt C Müller, Michael Bamberg, Kirsten Wachholz, Claus Belka, Verena Jendrossek

**Affiliations:** 1 Department of Radiooncology, University Hospital Tübingen, Hoppe-Seyler-Str. 3, 72076 Tübingen, Germany; 2 Department of Medicine III, University Hospital Grosshadern, Ludwig-Maximilians-University, Marchionistr.15, 81377 München, Germany; 3 Helmholtz-Zentrum München, Institute for Molecular Immunology, 81377 München, Germany; 4 Department for Clinical Chemistry, University Hospital Grosshadern, Ludwig-Maximilians-University, Marchionistr.15, 81377 München, Germany; 5 Max-Planck-Institute for Biophysical Chemistry, Am Fassberg 11, 37077 Göttingen, Germany; 6 Department of Radiooncology, University Hospital Grosshadern, Ludwig-Maximilians-University, Marchionistr. 15, 81377 München, Germany; 7 Department of Molecular Cell Biology, Institute of Cell Biology (Cancer Research), University of Duisburg-Essen Medical School, Virchowstr. 173, 45122 Essen, Germany

## Abstract

**Background:**

Alkylphosphocholines represent promising antineoplastic drugs that induce cell death in tumor cells by primary interaction with the cell membrane. Recently we could show that a combination of radiotherapy with Erufosine, a paradigmatic intravenously applicable alkylphosphocholine, *in vitro *leads to a clear increase of irradiation-induced cell death. In view of a possible combination of Erufosine and radiotherapy *in vivo *we determined the pharmacokinetics and bioavailability as well as the tolerability of Erufosine in nude mice.

**Methods:**

NMRI (nu/nu) nude mice were treated by intraperitoneal or subcutaneous injections of 5 to 40 mg/kg body weight Erufosine every 48 h for one to three weeks. Erufosine-concentrations were measured in brain, lungs, liver, small intestine, colon, spleen, kidney, stomach, adipoid tissue, and muscle by tandem-mass spectroscopy. Weight course, blood cell count and clinical chemistry were analyzed to evaluate general toxicity.

**Results:**

Intraperitoneal injections were generally well tolerated in all dose groups but led to a transient loss of the bodyweight (<10%) in a dose dependent manner. Subcutaneous injections of high-dose Erufosine caused local reactions at the injection site. Therefore, this regimen at 40 mg/kg body weight Erufosine was stopped after 14 days. No gross changes were observed in organ weight, clinical chemistry and white blood cell count in treated compared to untreated controls except for a moderate increase in lactate dehydrogenase and aspartate-aminotransferase after intensive treatment. Repeated Erufosine injections resulted in drug-accumulation in different organs with maximum concentrations of about 1000 nmol/g in spleen, kidney and lungs.

**Conclusion:**

Erufosine was well tolerated and organ-concentrations surpassed the cytotoxic drug concentrations *in vitro*. Our investigations establish the basis for a future efficacy testing of Erufosine in xenograft tumor models in nude mice alone and in combination with chemo- or radiotherapy.

## Background

Radiotherapy and chemotherapy are crucial components of most current protocols for the treatment of solid human tumors. Important mechanisms of antineoplastic action of these genotoxic therapies include induction of cell death, e.g., apoptosis or necrosis, and senescence. Unfortunately, tumorigenesis is characterized by tumor cells' evasion of cell death induced by oncogene activation or by conditions of stress in their specific environment. Because stress-induced and therapy-induced cell death share common cellular pathways, the same genetic alterations that mediate death resistance during carcinogenesis can cause cross-resistance to genotoxic therapies. Thus, targeting cell death resistance is a promising approach towards increasing the efficacy of genotoxic therapies for human solid tumors [1-4].

Alkylphosphocholines (APC) represent promising antineoplastic agents with a particular mechanism of action: In contrast to standard chemotherapy and irradiation these synthetic phospholipid derivatives target cellular membranes and interfere with membrane lipid composition and the formation of lipid second messengers, thereby affecting the growth, cell cycle progression, and survival of tumor cells without direct interaction with cellular DNA [5,6]. The antineoplastic action of synthetic phospholipid analogs relies on their ability to affect specific signaling processes in the target cells. Until now, the PI3K/Akt pathway, the mitogen-activated protein kinase (MAPK)/extracellular signal-regulated kinase (ERK) pathway, the stress activated protein kinase (SAPK)/Jun-N terminal kinase (JNK) and the sphingolipid pathway have been identified as important drug targets [7-9]. Moreover, APC with antineoplastic activity, e.g. Miltefosine, Perifosine, and Erufosine, induce apoptosis in tumor cells *in vitro*. Depending on the cell type, the induction of apoptosis involves ligand-independent activation of the death receptor pathway in membrane rafts, p53-independent activation of the mitochondrial apoptosis pathway, or both [7,8,10-12]. In contrast, induction of apoptosis by DNA-damaging agents (e.g. 5-fluorouracil) and irradiation is mainly dependent on p53-induced up-regulation of the pro-apoptotic Bcl-2 analog Bax. Interestingly, APC such as Miltefosine and ether lysolecithins such as Edelfosine increase the efficacy of chemotherapy and radiotherapy *in vitro *and in animal experiments [6,13]. These observations suggest that APC may be particularly useful for the treatment of tumor cells resistant to DNA-damaging drugs and irradiation.

The clinical use of the first generation APC Miltefosine was restricted to topical application due to hemolytic and gastrointestinal toxicity upon intravenous and oral application, respectively [14,15]. Furthermore, clinical trials testing the oral analogue Perifosine also revealed dose limiting gastrointestinal toxicity. The maximum tolerated dose after oral administration amounted to 200 mg/d for 3 weeks [16] and a maintenance dose of 100 mg/d could be achieved [17].

Erucylphosphocholine (ErPC), an APC derivative with a 22 carbon chain and a cis-double bond in the (omega-9)-position, lacks hemolytic toxicity due to the formation of lamellar instead of micellar structures in aqueous solutions and is therefore suitable for intravenous administration. In a first *in vivo *study in healthy rats, repeated intravenous injections of ErPC were well tolerated and revealed an accumulation of ErPC in different tissues, including brain [18]. However, *in vivo *application of ErPC was complicated by poor drug solubility in aqueous solutions due to gel formation. An intensive search for structural analogues with improved solubility properties resulted in Erufosine (ErPC3, Erucylphosphohomocholine). The structure of Erufosine in comparison to ErPC is characterized by the addition of one methylene group into the polar phosphocholine head group. Erufosine forms clear solutions in water and has similar antineoplastic activity *in vitro *[19].

To gain insight into the value of the novel APC derivative, Erufosine, in tumor therapy using mouse models, here we analyzed pharmacokinetics and biodistribution in nude mice after repeated intraperitoneal and subcutaneous drug application.

## Methods

### Chemicals

Erufosine (ErPC3, MG 503.8) is the (N,N,N-trimethyl)-propylammoniumester of erucyl-phosphoric acid. It was first synthesized by H. Eibl, Max Planck Institute of Biophysical Chemistry, Goettingen, Germany [20] and kindly provided for these studies. 1,2-Propanediol was purchased by Merck, Darmstadt, Germany. All other chemicals were from Sigma-Aldrich, Deisenhofen, Germany, if not otherwise indicated.

For aqueous solutions Erufosine was dissolved at 60°C in a mixture of distilled water and 1.2-Propandiol (mixture 98:2) to a final concentration of 24 mg/ml (48 mM) Erufosine and stored at 5°C after sterile filtration. For intraperitoneal and subcutaneous injection this stock solution was diluted with 0.9% sodium-chloride solution in the appropriate ratio to obtain the required dosage of Erufosine in the injection volume of 100 μl for 30 g mice. Differences in body weight of the mice were adjusted with injection volume.

### Animals

Animal experiments were made according to German animal welfare regulations and approved by the local authorities (registration number RO 1/05, Regierungspräsidium Tübingen). Immunodeficient NMRI-(nu/nu)-nude mice were purchased from the Central animal facility of the University of Duisburg Essen Medical School (age 4 months). Animals were housed in an individually ventilated cage rack system (Techniplast, Italy). They were fed with sterile high caloric laboratory food (Sniff, Germany). Drinking water was supplemented by chlorotetracycline and potassium sorbate acidified to a pH of 3.0 with hydrochloric acid and provided *ad libitum*. Mice were treated by intraperitoneal or subcutaneous injections of Erufosine every 48 h at the indicated drug concentrations for the biodistribution and toxicity studies or by a single intraperitoneal bolus injection for analysis of pharmacokinetic parameters in the serum. Intraperitoneal and subcutaneous drug injections were selected instead of intravenous drug application, as this application route is already well established for rodent models. Moreover, the experiments performed in the present study constitute the basis for future experiments designed to evaluate the antineoplastic action of Erufosine in combination with radiation. Subcutaneous and intraperitoneal administration is more practicable for the high numbers of animals bearing xenograft tumors that are required for those experiments.

Blood withdrawal was done by retro-orbital puncture in light diethylether anesthesia. Serum was obtained by centrifugation (5000 rpm, Eppendorf) and directly frozen at -20°C until analysis. Clinical chemistry was analyzed with standard protocols in the Central laboratory of the University Hospital Tübingen using ADVIA 1650 (Siemens, Eschborn). Blood cell count was done with ADVIA 120/2120 Cell counters (Siemens, Eschborn) from EDTA-blood.

For the biodistribution studies, brain, lungs, liver, stomach, spleen, kidney, first 5 cm of intestine, complete colon, muscle, and adipoid tissue were removed after blood withdrawal and immediate cervical distortion. The organs were weighed and stored at -20°C until analysis.

For the analysis of Erufosine-excretion, 12 mice were kept in single metabolic cages (Techniplast, Italy) with free access to food and water allowing urine sampling for the last 4 days of a two week treatment period. After an adaptation period of one day, urine was collected every 24-hours for 3 days under water-saturated oil and stored at -20°C.

### Analysis of ErPC3 in body fluids and tissues

For the quantitative measurement of Erufosine in serum and tissue samples liquid chromatography-tandem mass spectrometry (LC-MS/MS) was employed with a deuterium labeled analogue (ErPC3-D9, MW 512.82) as internal standard. Details are described elsewhere [21].

Briefly, for serum analysis an aliquot of 50 μl of serum was spiked with 20 μl ethanol containing 20 mg/l ErPC3-D9 in a 2 ml test tube. After vigorous mixing and equilibration for 20 min at room temperature, 1 ml of methanol/acetonitrile 9:1 (v/v) was added for protein precipitation. After centrifugation for 15 min at 16,000 × g, the clear supernatant was diluted 1:9 (v/v) with methanol/acetonetrile 9:1 (v/v) and proceeded for LC-MS/MS analysis. For tissue analysis 1 ml methanol/acetonitrile 9:1 (v/v), spiked with 20 μl ethanol containing 20 mg/l ErPC3-D9, was added to 100 mg tissue in a 1.5 ml test tube. Homogenization was performed after addition of a single carbide bead (diameter 3 mm) for 3 × 5 min with 40 Hz in a TissueLyser (Qiagen GmbH, Hilden, Germany). A clear supernatant was collected after centrifugation (15 min, 16,000 × g), subsequently diluted 1:9 (v/v) with methanol/acetonitrile 9:1 (v/v), and then proceeded for LC-MS/MS analysis.

A short CN column (20 × 4 mm I.D., 5 μm particle size, Dr. Maisch GmbH, Ammerbuch, Germany) was used for sample pre-fractionation with 70% methanol and 30% 0.1% formic acid delivered isocratically at a flow rate of 0.9 ml/min as the mobile phase. Applying a post-column split of approximately 1:10 the eluate was transferred to a Waters Quattro Ultima Pt triple stage mass spectrometer run in the positive electrospray mode. Using multiple reaction monitoring the mass transition 504.4>139.1 of the target analyte and the mass transition 513.7>139.1 of the deuterated standard was recorded. The analytical run time was 4 min. For calibration drug free serum was spiked with Erufosine in methanol. Six point calibration was performed in all analytical series.

### Statistics

If not otherwise stated, data are expressed as arithmetic means ± SD (n ≥ 3). Statistical data analysis was performed by paired or unpaired t-test, where appropriate. P ≤ 0.05 was considered statistically significant.

The pharmacokinetic data obtained after single intraperitoneal injections were calculated according to a two-compartment model using JMP 7.0.1 (SAS Institute inc.) software for approximation fit of the concentration curves.

## Results

### Pharmacokinetics after single bolus injection

Three groups of 5-6 mice each were administered a single injection of Erufosine (40 mg/kg body weight) by intraperitoneal injection. Approximately 50 μl of blood was drawn by retro-orbital puncture at different time points in each group, and mice were euthanized after the last puncture (group 1: 15 min, 30 min, 1 hour, 2 hours; group 2: 30 min, 2 hours, 4 hours, 8 hours; group 3: 1 hour, 4 hours, 12 hours, 24 hours and 36 hours).

**Figure 1:**
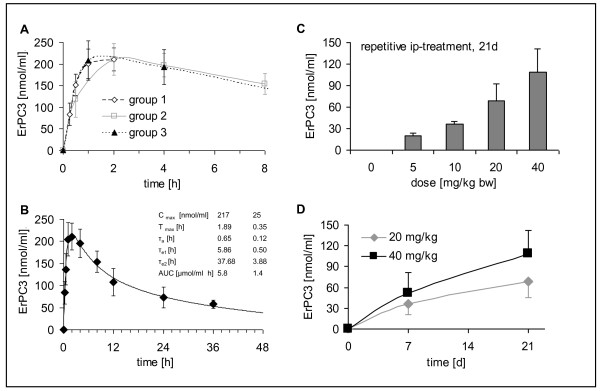
**Serum concentrations of Erufosine after a single bolus injection (A+B) or repeated injections (C+D)**. **A+B**: NMRI nu/nu mice were treated with one intraperitoneal injection of 40 mg/kg body weight Erufosine and subdivided into three groups for blood collection at different time points: group 1, n = 6 (◇): 15 min, 30 min, 1, 2 hours; group 2, n = 5 (□): 30 min, 2, 4, 8 hours; group 3, n = 5 (black triangle): 1, 4, 12, 24 and 36 hours. Erufosine concentrations in serum were determined by LC-MS/MS analysis. Data represent means ± SD: **A**. Data show the initial serum concentrations of groups 1-3 separately. **B**. Data show mean Erufosine serum-concentrations for all animals from group 1-3 pooled (n = 16). Insert shows the pharmacokinetic parameters. **C+D**: NMRI nu/nu mice were treated with repeated intraperitoneal injections of Erufosine every 48 hours at the indicated concentrations. All values are means ± SD (n = 3-6). Erufosine concentrations in serum were determined by LC-MS/MS analysis. **C**. Concentration-dependent increase in the serum levels of Erufosine after a three weeks treatment with 5, 10, 20 and 40 mg/kg body weight Erufosine. **D**. Time course of the Erufosine serum concentrations after treatment with 20 and 40 mg/kg body weight Erufosine for one and three weeks.

The highest serum concentrations upon single bolus ip-injection of 40 mg/kg body weight Erufosine, were measured 1 or 2 hours after treatment and achieved concentrations of 211 ± 27 nmol/ml (group 1, 2 h), 210 ± 36 nmol/ml (group 2, 2 h) and 209 ± 45 nmol/ml (group 3, 1 h). 36 hours after injection the serum concentration still had a value of 56 ± 12 nmol/ml (Fig. 1A+B). Because of high reproducibility among the three independent groups, the serum concentrations of all time points were averaged among the three groups. From these values we generated an approximation fit using an equation which consisted of a fast and a slow exponential decay combined with an exponential increase of serum levels (f(x) = A*(1-e^(-x/T)^)*e^(-x/T2)^+A*(1-e^(-x/T1)^)*e^(-x/T3)^). The quality of the fit was extremely good reaching a R^2 ^of 0.99 (Fig. 1A+B).

We further used this equation to calculate the pharmacokinetic parameters by fitting the serum values of each single mouse. For the animals with a short observation period (group1) we set the time constants for the decrease fix at the value pooled for all mice (Insert Fig. 1B).

Taken together, a single bolus injection of 40 mg/kg body weight Erufosine resulted in detectable serum concentrations over 36 hours with a maximum concentration of 217 ± 25 nmol/ml at 113 ± 20 min after injection.

### Repetitive injection

To study biodistribution of Erufosine, four different Erufosine-concentrations (5, 10, 20 and 40 mg/kg body weight) were administered every 48 h over a period of 7 (group 1), 14 (group 2) or 21 days (group 3) by intraperitoneal or subcutaneous injection. Each of the resulting 24 groups consisted of 3 to 9 mice. At the end of the treatment course 24 hours after the last injection approximately 300 μl blood were drawn and organs were removed as described above.

### Serum concentrations

The repetitive injection of Erufosine resulted in a concentration- and time-dependent increase in serum Erufosine-levels. After three weeks of intraperitoneal treatment with 5, 10, 20 and 40 mg/kg body weight Erufosine every 48 hours, respective serum concentrations amounted to 20 ± 4 nmol/ml, 36 ± 4 nmol/ml, 68 ± 23 nmol/ml and 109 ± 33 nmol/ml (Fig. 1C). Similar observations were made with subcutaneous injections (data not shown). The slope of the increase in serum concentrations was more pronounced in the first 7 days compared to longer treatment periods suggesting a convergence to steady state levels after prolonged Erufosine-treatment (Fig. 1D).

### Organ concentrations

**Figure 2:**
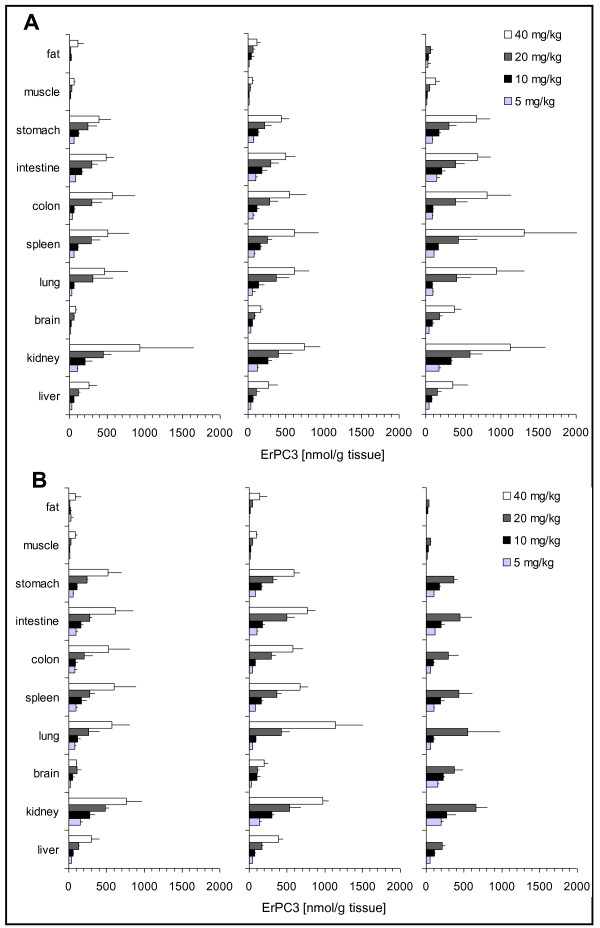
**Biodistribution of Erufosine after repeated drug injections**. Mice were separated into 24 groups and treated every 48 hours with a intraperitoneal or subcutaneous injection of Erufosine at the indicated concentrations for one, two or three weeks. At the end of the treatment period mice were killed, organs removed and organ concentrations of Erufosine were determined by LC-MS/MS analysis. All values are means ± SD (n = 3-9). **A**. Organ concentrations of Erufosine after intraperitoneal treatment with 5, 10, 20 and 40 mg/kg body weight Erufosine for one (left panel), two (middle panel) or three weeks (right panel). **B**. Organ concentration of Erufosine after subcutaneous treatment with 5, 10, 20 and 40 mg/kg body weight Erufosine for one (left panel), two (middle panel) or three weeks (right panel). Three weeks subcutaneous treatment with 40 mg/kg body weight Erufosine is missing due to local toxicity.

In a next step we analyzed the organ distribution of Erufosine in the three treatment groups after 7, 14, and 21 days of treatment (Fig. 2). Erufosine accumulated in all tissues included in the study. Maximum drug-concentrations were obtained after 21 days of intraperitoneal treatment in spleen (1307 nmol/g), kidney (1123 nmol/g) and lungs (939 nmol/g) (Fig. 2A). The respective subcutaneous injections led to slightly higher organ-concentrations at all concentrations used (Fig. 2B).

**Figure 3:**
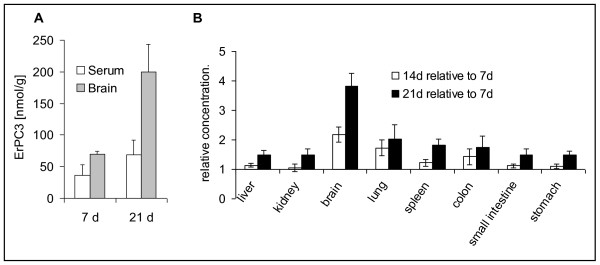
**Accumulation of Erufosine in brain tissue after repeated intraperitoneal drug injections**. Mice were treated every 48 hours with intraperitoneal injections of Erufosine at the indicated concentrations for one, two or three weeks. At the end of the treatment period mice were killed, organs removed and organ concentrations of Erufosine were determined by LC-MS/MS analysis. **A**. Brain and serum concentrations of Erufosine after treatment with 20 mg/kg body weight of Erufosine for 7 d and 21 d, respectively. Data show means ± SD (n = 3-6). **B**. Mean organ concentrations of Erufosine after treatment with 5, 10, 20 or 40 mg/kg body weight for 14 or 21 days were divided by the mean organ concentrations after the respective treatment for 7 days. Data show means ± SEM of the resulting quotients from all 4 dose groups (n = 12-24).

Because of a possible use of Erufosine for the treatment of glioblastoma, we were then interested in the drug concentrations that could be obtained in the brain tissue. Although absolute drug concentrations in the brain tissue were low compared to e.g. lungs or kidney, we could clearly demonstrate an increase of the brain/serum ratio after 7 and 21 days of treatment from 1.9 to 2.9, respectively, pointing to an accumulation of Erufosine in brain tissue (Fig 3A). With regard to the organ concentrations achieved after 14 and 21 days of treatment relative to the 7 day treatment, the most prominent time-dependent increase in the Erufosine-concentration was observed for brain tissue at all drug concentrations used (Fig 3B). It clearly demonstrates that Erufosine penetrates the blood-brain-barrier and accumulates in the brain tissue more efficiently compared to the other organs. The concentration in brain tissue after 3 weeks of treatment with 40 mg/kg body weight amounted to 383 nmol/g, which is clearly above the concentration required to induce death of glioblastoma cells *in vitro*.

### Urine excretion

**Figure 4:**
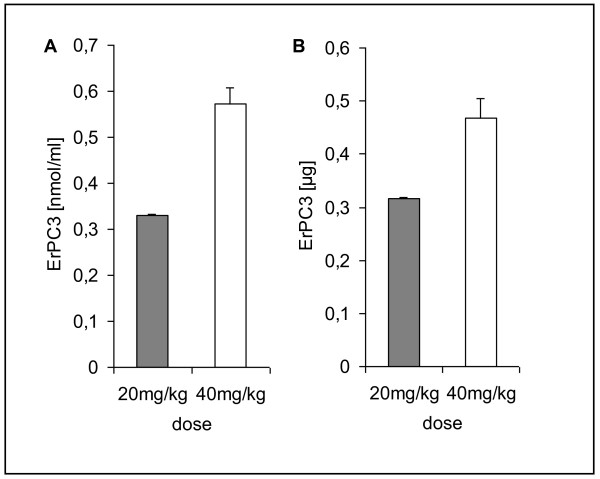
**Urine excretion of Erufosine after repeated intraperitoneal drug injections**. NMRI nu/nu mice were treated with intraperitoneal injection of 20 mg/kg body weight and 40 mg/kg body weight (n = 6 each) Erufosine every 48 hours for two weeks. The urine was collected over 24-hours on the last three consecutive days of the treatment period in a metabolic cage. Average urine volumes were determined and concentrations of Erufosine in urine were measured by using LC-MS/MS analysis. Data show (**A**) the urine concentrations and (**B**) the total amount of Erufosine (means ± SEM).

The average 24-hour urine excretion of Erufosine was measured for 6 mice during the last 3 consecutive days of a 14-day treatment period with intraperitoneal injections of 20 mg/kg or 40 mg/kg body weight Erufosine, respectively (Fig. 4). The average urine volume in both groups was comparable. Total quantity and concentration of Erufosine in the urine was very low yielding less than 0.5 μg and 0.6 nmol/ml Erufosine after treatment with 40 mg/kg body weight (Fig. 4). Taking into account that the serum concentrations was 64 nmol/ml and 122 nmol/ml Erufosine after a 14-day treatment with 20 mg/kg and 40 mg/kg body weight, the urine/serum ratio in both groups was less than 0.6%. Despite high absolute tissue concentrations in the kidney this demonstrates negligible urine excretion of Erufosine.

### Toxicity

**Figure 5:**
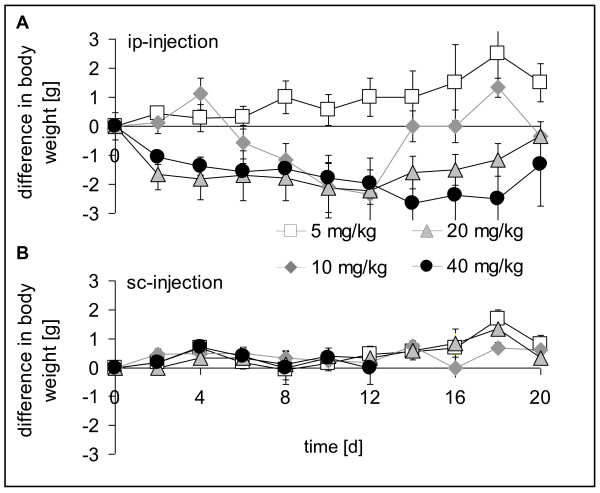
**Change in body weight of animals upon Erufosine treatment**. NMRI nu/nu mice were treated with intraperitoneal injections of 5, 10, 20 und 40 mg/kg body weight Erufosine every 48 hours. Body weight was determined every second day. Values represent means ± SEM of the difference from starting weight in the respective dose groups. **A**. Body weight after intraperitoneal injections. **B**. Body weight after subcutaneous injections.

Intraperitoneal injections of Erufosine were generally well tolerated. A clinical side effect of the high dose intraperitoneal treatment (40 mg/kg body weight) was transient diarrhea. No local changes or signs of inflammation were seen at the puncture. As an index of systemic toxicity the body weight of the mice was measured regularly. Mean weight of all animals at the beginning of treatment was 35.0 ± 1.2 g. Intraperitoneal application of 5 mg/kg body weight Erufosine did not result in any change of the body weight, whereas administration of higher concentrations led to a transient weight loss of less than 10% of body weight (Fig. 5).

In contrast, subcutaneous injections of Erufosine did not cause changes in body weight for all drug-concentrations used (Fig. 5). However, a local inflammation to the point of ulceration occurred at the puncture region after 14 d of treatment with 40 mg/kg body weight Erufosine (not shown). Therefore, the subcutaneous treatment with the high Erufosine concentration was stopped after 14 days.

At the end of the treatment course there were no macroscopic signs of organ injury and no systematic changes in organ weight (data not shown). Regarding the hematological parameters no bone marrow related toxicity was detectable even though the variance of white blood cell count was high.

The platelet count raised from 566 ± 155 for the control group to 833 ± 172 after 14 d treatment with 40 mg/kg body weight, but then decreased again until day 21 of the high dose treatment (Tab. 1). As shown in table 1, long-time treatment with 40 mg/kg body weight Erufosine led to a 2 to 2.5-fold increase of serum lactate dehydrogenase (LDH) after 14 and 21 days of treatment. Moreover, aspartate-aminotransferase (AST) was increased after 21 day treatment with 40 mg/kg body weight Erufosine, suggesting that high Erufosine-concentrations or long term treatment may induce some cell damage. However, no further significant changes in clinical chemistry and clinical picture could be detected arguing against a major toxic effect (Tab. 1). Certainly it has to be taken into account that nude mice can provide only a limited toxicity profile, in particular related to toxic immune responses.

**Table 1:** Serum parameters and hematological parameters during intraperitoneal Erufosine treatment (Mean ± SD)

		**14-d treatment**		**21-d treatment**	
	**control**	**20 mg/kg bw**	**40 mg/kg bw**	**20 mg/kg bw**	**40 mg/kg bw**

Blood count					

Leukocytes (/μl)	4,2 ± 2,1	3,3 ± 1,8	3,5 ± 2,5	5,0 ± 3,4	2,6 ± 1,4

Erythrocytes (10^6^/μl)	9,1 ± 0,4	8,4 ± 0,3	9,1 ± 0,8	9,1 ± 0,5	8,4 ± 1,4

Platelets (10^3^/μl)	566 ± 155	741 ± 94	833* ± 172	872* ± 432	442* ± 156

Hemoglobin (g/dl)	14,5 ± 0,6	13,6 ± 0,9	14,6 ± 1,9	14,0 ± 0,5	13,4 ± 1,9

Hematocrit (%)	47,5 ± 2,3	45,5 ± 1,7	49,1 ± 4,5	45,4 ± 4,7	44,7 ± 5,9

Serum					

Na (mmol/l)	156 ± 8	156 ± 2	155 ± 10	157 ± 4	162 ± 6

K (mmol/l)	5,1 ± 0,9	5,9 ± 1,2	6,3 ± 0,8	5,4 ± 0,8	5,8 ± 1,0

Ca (mmol/l)	2,4 ± 0,2	2,5 ± 0,1	2,6 ± 0,2	2,1 ± 0,3	2,5 ± 0,2

AST (U/l)	125 ± 42	117 ± 38	152 ± 22	140 ± 69	245* ± 156

ALT (U/l)	63 ± 32	55 ± 14	93 ± 25	65 ± 31	93 ± 45

LDH (U/l)	1315 ± 441	1401 ± 818	2650* ± 959	1913 ± 1152	3498* ± 519

Protein (g/dl)	5,1 ± 0,3	4,9 ± 0,1	5,5 ± 0,4	4,8 ± 0,3	4,9 ± 0,5

Creatinine (mg/dl)	0,3 ± 0,1	0,3 ± 0,1	0,3 ± 0,1	0,3 ± 0,1	0,3 ± 0,1

Urea (mg/dl)	56,5 ± 9,0	50,8 ± 8,1	57,3 ± 6,0	47,9 ± 6,4	56,2 ± 13,3

## Discussion

Here we show for the first time, that parenteral treatment of nude mice with Erufosine is feasible without major toxicity. Moreover, our data demonstrate that repeated intraperitoneal or subcutaneous injections of nontoxic Erufosine-concentrations yield organ concentrations that are sufficient to induce tumor cell death *in vitro*.

Tolerability of Erufosine-treatment was demonstrated by the absence of significant alterations in organ weight or macroscopic appearance, and minor changes in the body weight as an index of systemic toxicity. Only high dose intraperitoneal injection of Erufosine induced a mild diarrhea at the beginning of the treatment and a reversible weight loss preventing further dose escalation. These observations are reminiscent of earlier findings in healthy rats after high dose intravenous application of the Erufosine-related ErPC [18]. In contrast, subcutaneous application did not induce any changes in the body weight even upon treatment with 40 mg/kg body weight Erufosine. These observations suggest that intraperitoneal injection of Erufosine may induce a local effect similar to the gastrointestinal toxicity observed after oral application of Perifosine [13,16,17,22,23]. On the other hand, despite the absence of alterations in the body weight, subcutaneous injection was accompanied by dose limiting ulcerations at the injection site 2 weeks after treatment with 40 mg/kg body Erufosine. As prolonged intravenous infusion of low-dose Erufosine is well tolerated in patients (L. Lindner, personal communication) long-term intravenous infusion of Erufosine may be considered as an alternative for future experiments.

Clinical chemistry revealed a concentration-dependent increase in serum levels of LDH and to a lesser extent of AST during Erufosine-treatment, while alanine-aminotransferase and further blood parameters remained unchanged. Although the increase in LDH has been described as a hint for beginning hemolysis, being a major toxic side effect of the first generation APC Miltefosine [14], the lack of changes in the hemoglobin levels and of a clinical correlate argues against a clinically relevant hemolytic effect of Erufosine. It may be suggested that Erufosine-treatment affects the membrane composition of the erythrocytes facilitating damage of more fragile erythrocytes during retro-orbital blood withdrawal. Since a marginal elevation of AST-levels after intravenous application of ErPC in rats has been already described for the Erufosine related ErPC [18] this parameter should be further analyzed in preclinical or clinical trials.

Kidney related serum parameters like electrolytes, protein, creatinine and urea did not increase during treatment with Erufosine leaving no evidence for renal dysfunction as described for Miltefosine [24].

Importantly, similar to previous reports for other APC, Erufosine lacked bone marrow toxicity [18,24,25]. However, in contrast to earlier investigations with Miltefosine or ErPC, instead of the reported increase in leukocyte numbers, we only detected a time- and concentration-dependent transient increase in thrombocyte numbers. The differences in the blood cell behaviour may be related to the distinct application mode and/or species-specific differences in the drug effect.

A single bolus injection of Erufosine resulted in detectable serum Erufosine levels for approximately 36 h peaking at 217 ± 25 nmol/ml 113 ± 20 min after injection. Repeated intraperitoneal or subcutaneous administrations led to a continuous increase of serum and organ concentrations of Erufosine with the highest concentrations achieved in spleen, kidney and the lungs. The subcutaneous injections yielded slightly higher drug-concentrations in most tissues compared to the intraperitoneal injections reaching significance in liver, kidney, and brain. Our data corroborate earlier findings about the bioavailability of the Erufosine-related ErPC in healthy rats [18]. Although organ distributions were quite similar, the Erufosine-concentrations achieved upon intraperitoneal or subcutaneous administration in the respective tissues were increased in most of the tested organs compared to the ErPC-concentrations obtained after intravenous injections in the investigation of Erdlenbruch and coworkers [18]. This may at least partially be related to the altered serum composition observed in nude mice. Moreover, the increased sensitivity of liquid chromatography-tandem mass spectrometry used in the present study compared to that of high performance thin layer chromatography HPTLC used in the earlier investigation may be of relevance [21].

In contrast, Erufosine-concentrations in the brain tissue were below the levels obtained by Erdlenbruch et al. [18], an effect that may reflect distinct efficiency in crossing the blood brain barrier due to the distinct lipophilic behaviour of the two derivatives and/or altered composition of the blood brain barrier in rats compared to mice. Nevertheless, we observed a strong time- and concentration-dependent accumulation of Erufosine in the brain tissue reaching 383 nmol/g after a 3-week treatment with 40 mg/kg of body weight. This concentration is clearly above the concentration sufficient to induce cytotoxicity in malignant glioma cell lines *in vitro *[10,19,26,27].

Together with our earlier investigations on the increased cytotoxic efficacy of ionizing radiation in combination with Erufosine in glioblastoma cell lines *in vitro*, the ability of Erufosine to cross the blood brain barrier and to accumulate in the brain tissue make the drug a promising candidate for combined treatment approaches with radiotherapy in malignant glioma. Although clinical trials already demonstrated feasibility and tolerability of a therapy with Perifosine, Erufosine, or Perifosine with radiotherapy for patients with advanced human malignancies [[8,13] and personal communication with L. Lindner], before debarking into clinical trials with patients suffering from malignant glioma and other tumors, efficacy of Erufosine in combined treatment approaches has to be evaluated in animal experiments *in vivo*.

In conclusion, our data reveal that intraperitoneal and subcutaneous administration of Erufosine to nude mice is feasible and safe. Furthermore the concentrations achieved in the brain tissue are above the concentrations needed for combination effects with radiation in earlier *in vitro *experiments using human astrocytoma/glioblastoma cell lines. Our results constitute the basis for the design of preclinical investigations with Erufosine alone and in combination with radiotherapy in murine tumor models, in particular in nude mice. In a next step, we will evaluate efficacy of Erufosine in combination with ionizing radiation *in vivo *in nude mice bearing subcutaneous tumors. Based on our present investigations, pretreatment with repeated intraperitoneal injections of Erufosine for 1 or 2 weeks prior to initiation of radiotherapy should be considered to benefit from the drug-accumulation in the tumor tissue.

## Competing interests

The authors declare that they have no competing interests.

## Authors' contributions

GH contributed significantly to the design of the study, data acquisition, data analysis and drafting the manuscript. LHL contributed significantly to data acquisition, data analysis and drafting the manuscript. KW and MV performed probe preparation and mass spectrometry measurements, respectively. JW performed many of the animal experiments. ACM and MB performed critical revision of the manuscript. HE synthesized and provided ErPC and ErPC3 for the analysis. CB participated in the conception of the study and interpretation of data. VJ performed conception and design of the study and substantially contributed to interpretation of data, drafting of the manuscript, critical revision of the manuscript and final approval. All authors read and approved the final manuscript.
